# An Optical Fiber Sensor Coated with Electrospinning Polyvinyl Alcohol/Carbon Nanotubes Composite Film

**DOI:** 10.3390/s20236996

**Published:** 2020-12-07

**Authors:** Jinze Li, Xin Liu, Hao Sun, Liming Wang, Jianqi Zhang, Li Deng, Tianhong Ma

**Affiliations:** 1 School of Physics and Optoelectronic Engineering, Xidian University, Xi’an 710071, China; jinzeli@stu.xidian.edu.cn (J.L.); liuxin0327@stu.xjtu.edu.cn (X.L.); jqzhang@mail.xidian.edu.cn (J.Z.); ldeng@stu.xidian.edu.cn (L.D.); thma_1@stu.xidian.edu.cn (T.M.); 2 Wide Bandgap Semiconductor Technology Disciplines State Key Laboratory, School of Microelectronics, Xidian University, Xi’an 710071, China; lmwang@xidian.edu.cn

**Keywords:** optical fiber sensor, electrospinning, nanofiber film, humidity, temperature

## Abstract

A fiber-optics tapered sensor that is covered by an electrospinning polyvinyl alcohol (PVA) nanofiber film, is demonstrated to measure humidity and temperature simultaneously. A section multi-mode fiber (MMF) was sandwiched between two leading-in and out single mode fibers (SMFs), which was further tapered down to 29 μm to promote the humidity sensitivity of the sensor. A thin layer of electrospinning PVA nanofiber film was uniformly coated on the MMF taper region by electrospinning technology. In order to promote the humidity sensitivity and mechanical strength of electrospinning nanofibers, the carbon nanotubes (CNTs) were mixed into PVA to formed PVA/CNTs composite nanofiber film. A Fiber Bragg Grating (FBG) was cascaded with the humidity sensing fiber to monitor the ambient temperature simultaneously. The addition of CNTs effectively eliminated the cracks on the electrospinning nanofiber and made it more uniform and smoother. As experimental results show, the humidity sensitivity of the sensor with PVA/CNTs film was 0.0484 dB/%RH, an improvement of 31.16% compared to that of the sensor with PVA film, for which sensitivity is 0.0369 dB/%RH. The nanofiber humidity-sensitive film constructed using electrospinning had a satisfactory humidity response, special 3D structure and extensive application prospect.

## 1. Introduction

Humidity monitoring is particularly important in the industrial, agricultural, medical, chemical, and construction fields [1,2,3,4]. The demand for humidity sensors in various industries has recently promoted continuous research on different types of optical fiber humidity sensors [5]. It is necessary to develop a humidity sensor with high sensitivity and a high humidity response range. For certain narrow, intense electromagnetic radiation, high temperature and high-pressure environments, traditional humidity sensors will not work correctly. The optical fiber humidity sensor can overcome the influence of these extreme environments and work correctly. Therefore, the development of optical fiber humidity sensors has received increasingly more attention.

The multi-mode interference-based optical fiber sensing structure is highly sensitive to the ambient refractive index change; hence, it is widely developed as a refractometer by probing the cladding mode perturbation [6,7]. Because the silica fiber itself is insensitive to ambient humidity change, merging the humidity-sensitive materials and optical fiber are the traditional methods to fabricate the highly sensitive humidity sensors. The materials commonly used with optical fiber humidity sensors include polymethyl methacrylate (PMMA) [8], chitosan [9], agarose [10], calcium alginate [11], polyimide (PI) [12], carboxymethyl cellulose (CMC) [13], PVA [14]. Among them, PVA is a high molecular polymer widely used in optical fiber humidity sensors. PVA is a water-soluble polymer with a semi-crystalline structure. PVA has good hygroscopicity, biocompatibility, chemical stability, and is easily soluble in water [15]. In addition, it also has good fiber-forming, film-forming, and cohesive properties [16]. PVA has a wide range of applications in chemical fiber, medicine, and food industries [17]. Because of these advantages, many optical fiber humidity sensors choose PVA as the humidity-sensitive material [18].

The tapered optical fiber sensor has high sensitivity, compact structure, wide dynamic response range, and low cost. Therefore, there are many tapered sensors, such as refractive index sensors [19], biochemical sensors [20], strain sensors [21], pressure sensors [22], and temperature sensors [23]. When the incident light is transmitted to the tapered area, the core of the tapered fiber becomes thinner, which causes the mode field radius of the core guided mode to change, which transforms into the cladding transmission at the tapered waist. When a layer of humidity-sensitive material is coated on the taper waist, the refractive index of humidity-sensitive material changes with the humidity changes, resulting in a change in the output light’s optical power. Optical fiber sensors generally need to be combined with humidity sensitive materials to have highly sensitive characteristics. 

The film-forming methods usually used are vacuum evaporation [24], ion sputtering coating [25], sol-gel technology [26], layer-by-layer self-assembly (LBL) [27], and the dip-coating method [28]. Compared with these methods, electrospinning is a simple and effective method to form a layer of nanofiber film on the surface of the optical fiber. Electrospinning refers to the process of forming nanofibers through the action of a high-voltage electric field when the polymer is melted or dissolved. First, the polymer solution is charged with a certain amount of electrostatic charge under the high-voltage static electricity field. Second, the liquid droplets form a Taylor cone of the capillary under the interaction of the electric field force and the surface tension of the solution. Then, with the increase of voltage, the smaller droplets with dots are accelerated at the apex of the needle tip. Finally, when the electric field force is large enough to overcome the surface tension constraint, polymerization forms droplets of the spray, emitting a thin stream. The thin stream will eventually form a nanofiber film on the grounded receiving device as the solvent evaporates or solidifies in the spraying process. Electrospinning is a technology available that can be used to continuously prepare fibers with a diameter as low as a few nanometers. The nanofiber diameter prepared by electrospinning is nano grade with a diameter typically in the range of 3nm-5μm [29]. The nanofiber processed by electrospinning has a larger specific surface area compared to conventional coating film, which can absorb a large number of water molecules. However, there are relatively few studies on optical fiber humidity sensors using the electrospinning nanofiber film.

CNTs have attracted great interest owing to their unique structure, mechanical properties, and electrical properties [30]. And it has made significant breakthroughs in many research fields, such as nanoelectronics devices, high-strength composite materials, sensors, catalysts, and biomolecular carriers. CNTs are very suitable for absorbing water molecules or other gas molecules because CNTs have a large surface area to volume ratio, enable their use for humidity detection. 

In this work, an optical fiber sensor was designed for measuring temperature and humidity simultaneously, using tapered MMF cascaded FBG. Unlike traditional optical fiber coating methods, electrospinning is used to construct a humidity-sensitive nanofiber film quickly and simply. Experimental tests show that the produced electrospinning nanofiber film sensor is sensitive to temperature and humidity, and so electrospinning nanofiber film has good application prospects in future optical fiber sensors.

## 2. Sensor Manufacture

### 2.1. Sensor Fabrication and Transmission Theory

#### 2.1.1. Sensor Structure Design and Manufacture

**Figure 1:**
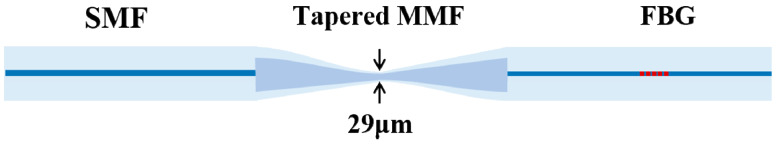
Schematic diagram of the sensor structure.

A schematic diagram of the sensing structure is presented in Figure 1. The fabricated optical fiber sensor is composed of a 1 cm tapered MMF and an FBG. In this paper, the tapered structure was fabricated using the pull taper function of the optical fiber fusion splicer: 5 mm-long MMF was spliced with a single mode fiber (SMF). Another 5 mm MMF was spliced to an SMF with an FBG inscription. The two MMFs were spliced together and tapered simultaneously under fusion conditions, a discharge electric strength of 60 bits, a discharge time of 190 μs, and an advancing speed of 0.75 mm/s. The core diameter of the MMF used was 105 μm and the cladding diameter was 125 μm. The center wavelength of the FBG used was 1550 nm. Then, a layer of electrospinning nanofiber film was coated on the optical fiber. Thus, an optical fiber sensor with electrospinning nanofiber film was manufactured.

#### 2.1.2. Tapered Fiber Transmission Theory

The field strength of the light wave in the optical fiber is:

(1) $$E_{m}{(r,\varphi ,z)=E}_{m}\left(r,\varphi )\exp[j(\beta z-\omega t)\right]\;\left(m=1,3\right)$$

where φ is the tangential parameter of the optical fiber, m=1 is the fiber core, m=3 is the fiber cladding, ω is the circular frequency and β is the propagation constant. 

The incident light field from SMF to MMF can be expressed as:

(2) $$E_{0}(r,0)=\sum_{m=1}^{n}a_{m}E_{m}(r)$$

where $E_{m}$ and $a_{m}$ are the eigenmodes field profile and excitation coefficient of each mode. After the light is transmitted for a certain distance in the MMF, the light field distribution is:

(3) $$E(r,L)=\sum_{m=1}^{n}a_{m}E_{m}(r)\exp(j\beta_{m}L)$$

Assuming that the input power of the SMF is $P_{in}$, the reflectivity of the FBG is $R_{FBG}$ and the output power $P_{out}$ is:

(4) $$P_{out}=10\log_{10}\left\{\frac{{\left|\int_{0}^{\infty }E(r,L)E_{0}(r)rdr\right|}^{2}}{\int_{0}^{\infty }{\left|E(r,L)\right|}^{2}rdr\int_{0}^{\infty }{\left|E_{0}(r)\right|}^{2}rdr}\right\}P_{in}R_{FBG}$$

From Equation (4), $P_{out}$ is mainly affected by the refractive index of the core of the MMF, the refractive index of the cladding of the MMF, and the length of MMF. For our sensor, the refractive index of MMF’s core and the length of the MMF were fixed, while the refractive index of the cladding of MMF changed after the nanofiber absorbs water. Therefore, changes in humidity cause changes in the sensor output power.

### 2.2. Preparation of PVA Gel Solution and PVA/CNTs Composite Solution for Electrospinning

PVA gel solution preparation: 8 g PVA (Xilong scientific, Shanghai, Chinna, AR) was added to 100 mL deionized water. A magnetic stirrer was used to stir the solution for 6 h at 50 °C and 2000 r/min until all the PVA powder was completely dissolved. The preparation of PVA/CNTs composite solution: 0.4 g CNTs (XFNANO.Inc, Jiangsu, China, multi-wall carbon nanotubes) were put into 100 mL deionized water, and then oscillated by an ultrasonic oscillator (Doves, 020s, Guangdong, China) for 1.5 h, resulting in uniform dispersion of the CNTs in water. Finally, 8 g PVA powder was added into the solution and stirred by a magnetic stirrer with 6 h at 50 °C and 2000 r/min until all the PVA powder was dissolved entirely and formed a uniform PVA/CNTs composite solution.

### 2.3. Electrospinning Nanofiber Film Manufacturing

The voltage, injection speed, type of needle tip, collector, collecting distance, and temperature in the electrospinning process will all affect the film-forming effect of the final nanofiber film.

#### 2.3.1. Voltage

**Figure 2:**
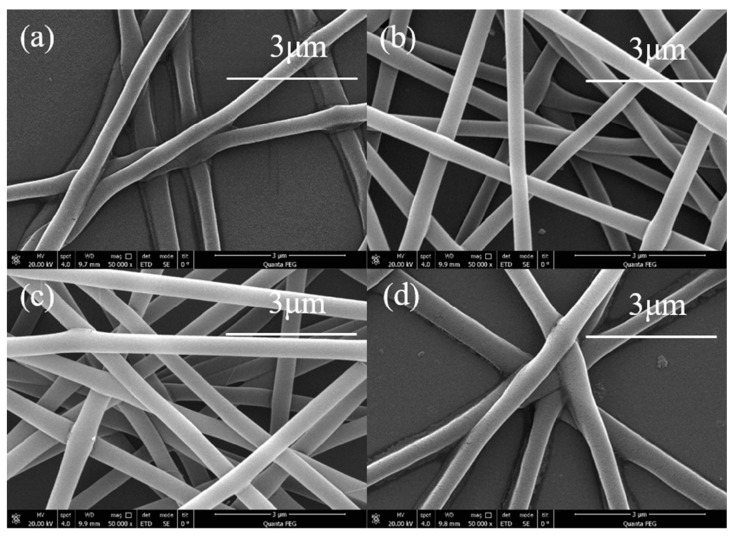
SEM image of PVA electrospinning nanofibers at different voltages. (**a**)14.5 kV (**b**) 16.5 kV (**c**) 18.5 kV (**d**) 22.5 kV.

Under normal circumstances, a higher voltage results in a greater tip jet’s Coulomb force, and it is easier for the droplets to stretch and extend, resulting in a smaller fiber diameter. However, when the additional voltage is too large, the amount of charge will accumulate rapidly, and the jet will be ejected very fast, which will cause the Taylor cone to be small and unstable, and block the spinning process. As shown in Figure 2, when the voltage is as low as 14.5 kV (Figure 2a) or as high as 22.5 kV (Figure 2d), the nanofiber diameter produced by electrospinning is not uniform enough. When the voltage is 16.5 kV (Figure 2b) and 18.5 kV (Figure 2c), relatively uniform and stable nanofibers can be produced. Therefore, in the current work, the electrospinning voltage was set to 18.5 kV. 

#### 2.3.2. Injection Speed

**Figure 3:**
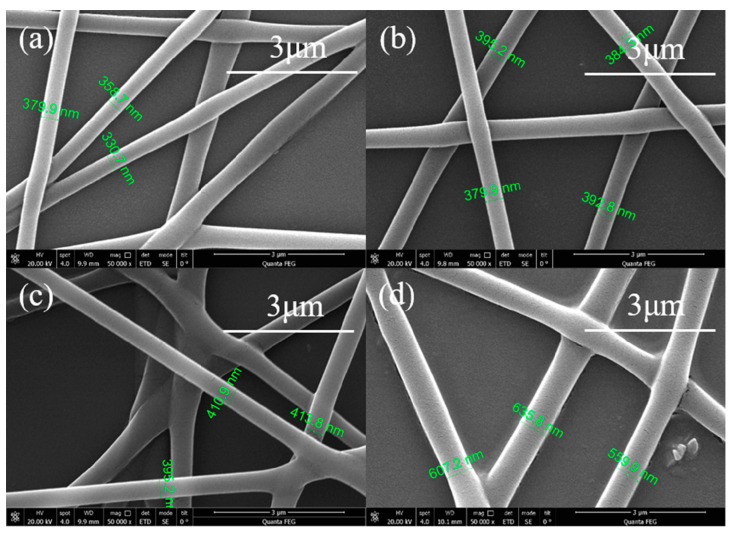
SEM image of PVA electrospinning nanofibers at different injection speed. (**a**) 0.0010 mm/s (**b**) 0.0015 mm/s (**c**) 0.0020 mm/s (**d**) 0.0025 mm/s.

The injection speed determines the supply amount of the available polymer solution. For each established voltage, a corresponding amount of injection is required to ensure the stability of the Taylor cone. Theoretically, when the injection volume increases, the solution volume extended by the needle tip increases, and the fiber diameter increases, as observed in Figure 3. Due to the rise in the volume of liquid ejected from the needle tip, the jet flow requires a longer time to dry. If the injection volume is too large, the solvent may not be fully dried and may form a solution solute accumulation layer instead of nanofibers, as observed in Figure 3c,d. Therefore, the appropriate injection volume can ensure that the solvent has sufficient volatilization time to form stable nanofibers and the injection speed selected in this work was 0.0015 mm/s.

#### 2.3.3. Solution Concentration

**Figure 4:**
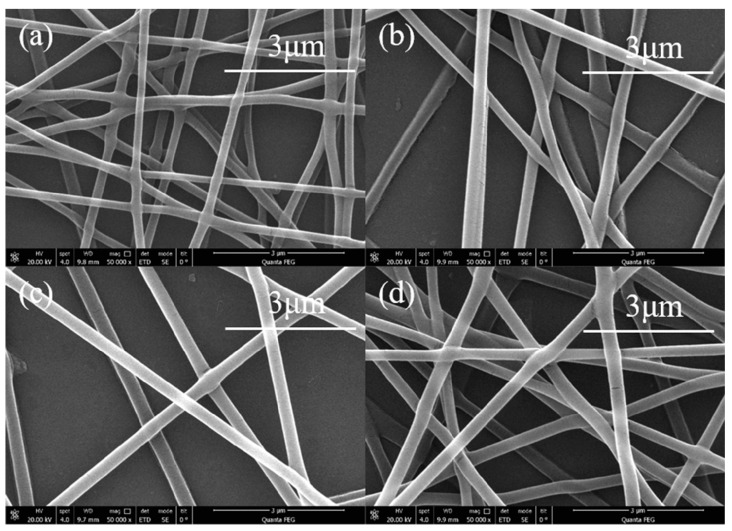
SEM image of PVA electrospinning nanofibers with different concentration. (**a**) 0.06 g/mL (**b**) 0.07 g/mL (**c**) 0.08 g/mL (**d**) 0.09 g/mL.

The change in the concentration of the high molecular polymer solution is its viscosity. Changes in viscosity affect the process of electrospinning. It can be observed from Figure 4a that the diameter of the electrospinning nanofibers was smaller when the PVA solution concentration was low, and the shape of the nanofibers was greatly distorted. As the solution concentration increased, the electrospinning nanofiber diameter and shape become uniform, as shown in Figure 4b,c. When the solution concentration was 0.09 g/mL, as shown in Figure 4d, the excessive concentration led in a greater the viscosity of the solution, hindering the flow of the solution and causing the formed fibers to bend or produce defects. In this work, we used 0.08 g/mL PVA solution to obtain better nanofibers.

#### 2.3.4. Preparation of Electrospinning Nanofiber Film

**Figure 5:**
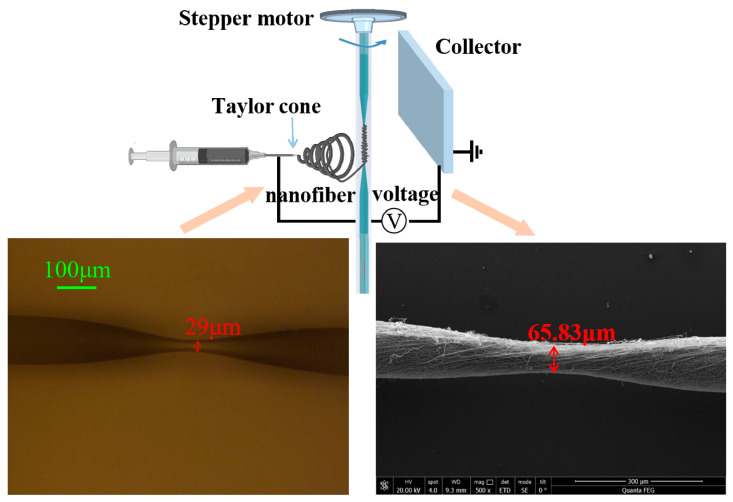
Schematic diagram of electrospinning process, the tapered MMF and the tapered MMF with PVA/CNTs composite nanofiber film.

The electrospinning machine model used in the experiment was DP30 (Yunfan Instrument). After many experiments, electrospinning was performed under a voltage of 18.5 kV, injection speed of 0.0015 mm/s, 18 G needle tip (inner diameter = 0.84 mm, outer diameter = 1.27 mm, length = 50 mm), collection distance of 14 cm, and temperature of 27 °C. Under these conditions, a better nanofiber film was obtained. Figure 5 presents a schematic diagram of the electrospinning process, the tapered MMF and the tapered MMF with PVA/CNTs composite nanofiber film. From the micrograph in Figure 5, we can see that the tapered fiber waist diameter is 29 μm and the length of the tapered area was 590 μm. After the sensor was covered with a layer of electrospinning PVA/CNTs composite film, the diameter of the tapered waist became 65.83 μm, which indicates that the thickness of the film was 18.42 μm.

**Figure 6:**
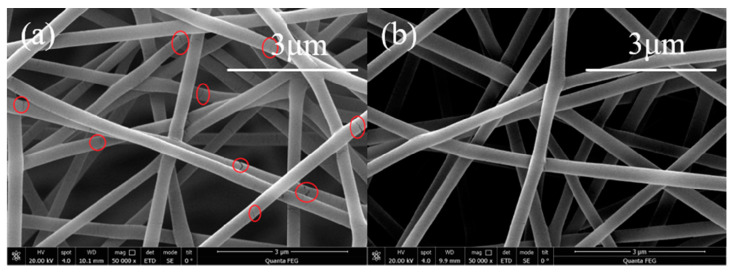
(**a**) PVA electrospinning nanofiber film and (**b**) PVA/CNTs composite nanofiber film.

A layer of uniform nanofiber film must be made in the tapered area of the optical fiber for humidity sensing. If the optical fiber is fixed on the collector for electrospinning, only the side close to the needle tip will have a nanofiber film, and the other side will have almost no nanofibers. This will affect the performance of the sensor. To solve this problem, we placed a stepper motor on the top of the electrospinning machine, and then fixed the optical fiber sensor on the stepper motor. The stepper motor drove the optical fiber sensor to rotate, and then the electrospinning fiber was able to form a layer of nanofiber film around the cone of the sensor. After the experiments, the speed of the stepper motor was set to 300r/min and the electrospinning time was set to 4 min. Under these conditions, a layer of satisfactory nanofiber film was obtained in the tapered area. The same method and settings were used to make optical fiber sensors based on PVA nanofiber film (as shown in Figure 6a) and PVA/CNTs composite nanofiber film (as shown in Figure 6b). The sensors were then placed in the drying box for 12 h such that the nanofiber film could be stably attached to the sensor. Finally, we performed temperature and humidity response analysis.

Alaa proved through experiments that after adding CNTs to polyacrylonitrile (PAN), the ultimate elastic modulus and tensile strength of the composite electrospinning nanofibers increased by 84% and 38%, respectively [31]. Dan proved that the tensile strength and elongation for the polyimide/CNTs composite electrospinning nanofibers compared with polyimide nanofibers were increased by 138% and 104% [32]. Ding showed that the mechanical parameters and conductivity as well as visibility to light of the films could be enhanced simultaneously by varying the concentration of the MWCNTs-COOH suspension and PVA solution [33]. Song used graphene oxide as the dispersing and added MWCNTs in the PVA solution, the results showing that the tensile strength of the nanofiber mat had increased [34]. Jeong prepared PVA and MWCNTs composite nanofibers and exhibited an enhancement in tensile strength from 5.8 MPa to 9.3 MPa compared to non-woven PVA [35]. As shown in Figure 6, comparing the photos of the PVA nanofiber film in Figure 6a and the PVA/CNTs nanofiber film in Figure 6b, it can be observed that there were many cracks in the PVA electrospinning fiber. However, there are almost no cracks in the PVA/CNTs composite nanofibers. Experimental results show that the addition of CNTs improves the humidity sensitivity of the PVA nanofiber film and increases the mechanical strength of the film.

## 3. Humidity and Temperature Experiments

**Figure 7:**
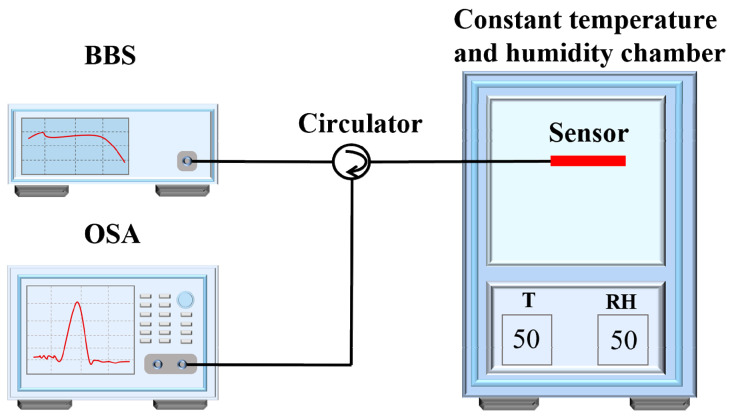
Schematic diagram of the temperature and humidity response experiment test.

Once the sensor was manufactured, its sensitivity responses to humidity and temperature were tested. The experimental setup consisted of a broadband light source (BBS, CL-17NBFA, Hoyatek), circulator, optical spectrum analyzer (OSA, AQ6370B, Yokogawa), and constant temperature and humidity chamber (BPS-50CL, Bluepard Instruments). A schematic diagram of the experimental setup is presented in Figure 7.

### 3.1. Humidity Response Test

**Figure 8:**
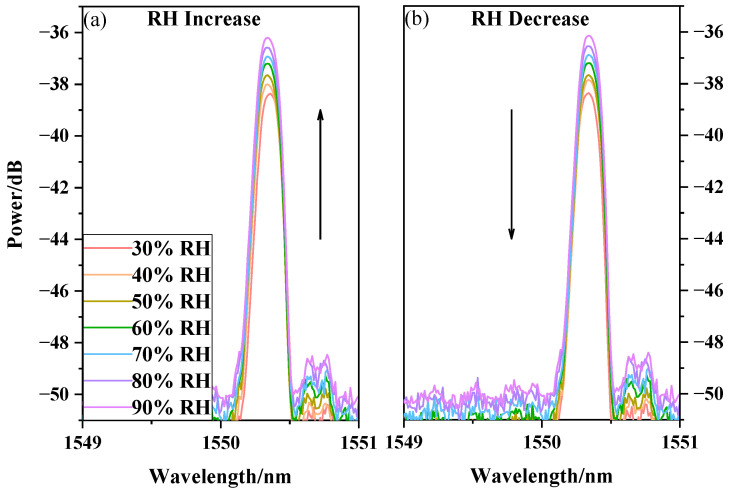
PVA nanofiber film humidity response with RH increase and decrease. (**a**) RH Increase; (**b**) RH Decrease.

The prepared sensor with the PVA nanofiber film was placed in a constant temperature and humidity chamber. In the process, the temperature and relative humidity of the chamber were set to 50 °C and 30%RH, respectively, and these conditions were maintained for 30 min to ensure a stable temperature and humidity ambience. The humidity range is from 30%RH to 90%RH, and the temperature was recorded at each 10%RH change. The output spectra for when the relative humidity slowly increased and decreased are presented in Figure 8a,b, respectively. The experimental results show that as the relative humidity increased, the PVA electrospinning nanofiber film absorbed water and changed the refractive index, leading to the changes in the optical waveguide in the tapered area, and thus the changes in optical power. In Figure 8, as the relative humidity increased, the output power of the sensor increased. Conversely, as the relative humidity decreased, the output power of the sensor decreased. Experimental results show that the PVA nanofiber film sensor’s response time from 30%RH to 90%RH is 2.53 s, and the response time from 90%RH to 30%RH is 2.67 s.

**Figure 9:**
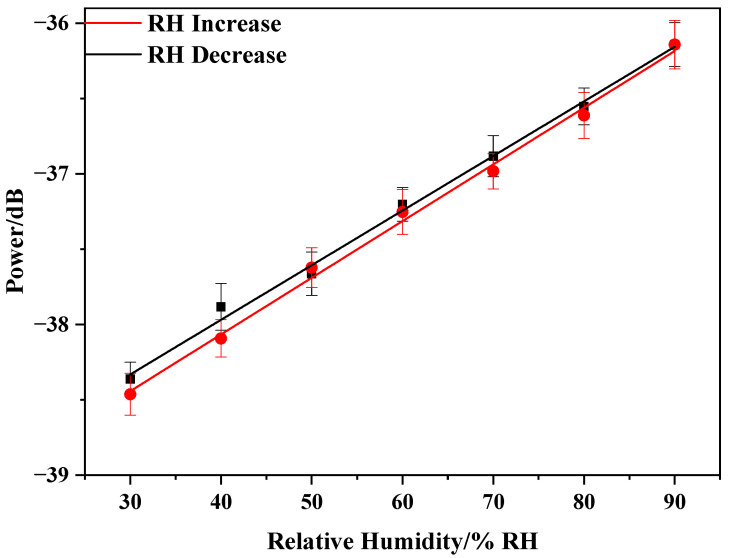
PVA nanofiber film humidity response fitting curve.

Figure 9 shows the relationship between the relative humidity changes and power changes for increasing and decreasing RH: y = 0.0377x − 39.573 and y = 0.0361x − 39.406. The sensor presents a linear response to RH with a sensitivity of 0.0369 dB/%RH. The power change of the sensor with the relative humidity is roughly linear with the relative humidity. The small difference primarily results from the humidity hysteresis of the PVA nanofiber film. To ensure the stability of the sensor, we repeated three cycles for each set of experiments. The proposed sensor showed good stability, as indicated by the standard deviation column in Figure 9. The fitting curve shows that the sensor was sensitive to humidity changes. Thus, the PVA electrospinning nanofiber film can be used as a new sensitivity coating for optical fiber sensors.

However, it can be observed from the SEM images of the PVA nanofibers that some of the PVA electrospinning nanofibers had some cracks (observed in Figure 6). This finding indicates that the mechanical strength of the PVA nanofibers was not very ideal and easily led to nanofiber film collapse. Related studies have shown that adding CNTs to an electrospinning polymer solution can improve the nanofiber mechanical strength. In addition, the CNTs have a large specific surface area and can absorb more water molecules. Therefore, we fabricated a PVA/CNTs composite nanofiber film to improve the mechanical strength, and sensitivity and studied the sensor response characteristics using the composite film.

**Figure 10:**
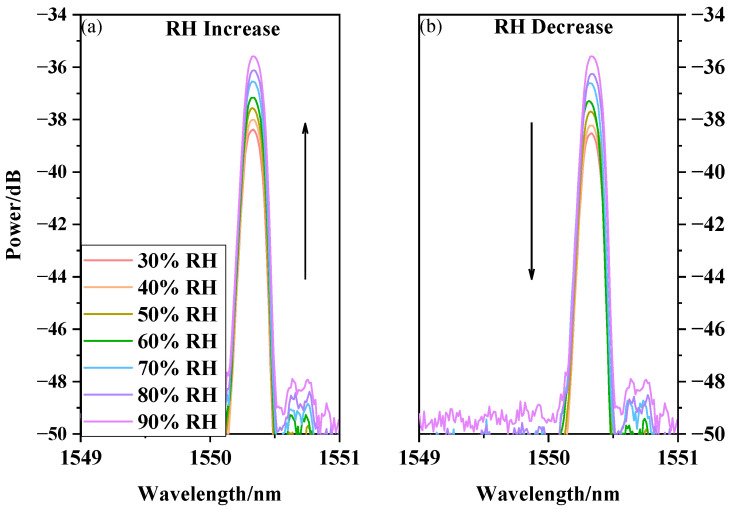
PVA/CNTs composite nanofiber film humidity response with RH increase and decrease. (**a**) RH Increase; (**b**) RH Decrease.

**Figure 11:**
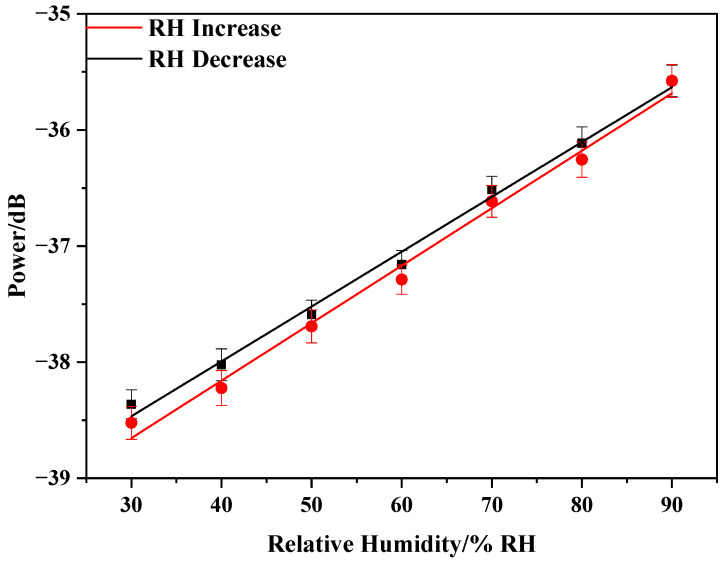
PVA/CNTs composite nanofiber film humidity fitting curve.

After testing the sensor humidity response with the PVA nanofiber film, we further performed the same humidity response test of the sensor with the PVA/CNTs composite nanofiber film to study the effect of CNTs on the humidity sensing. The experimental results are presented in Figure 10a,b. The sensor power change as a function of the relative humidity was roughly linear, with y = 0.0495x − 40.135 and y = 0.0473x − 39.888 as RH increased and decreased. The sensor exhibited a linear response to RH with a sensitivity of 0.0484 dB/%RH. Similarly, to improve the accuracy of the experimental test data, we performed three repeated experiments. According to the standard deviation column in Figure 11, the sensor also had good stability. The experimental results show that after adding CNTs, the sensor humidity sensitivity with the electrospinning nanofiber film increased by approximately 31.16%. For the PVA/CNTs composite film sensor, the response time from 30%RH to 90%RH is 2.44 s and from 30%RH to 90%RH is 2.55 s, respectively.

The experimental results indicate that after adding CNTs, the sensor sensitivity improved, and in addition, the mechanical strength of the electrospinning nanofiber on the sensor improved. These changes contributed to the sensor working more sensitively and stably.

### 3.2. Temperature Response Test

**Figure 12:**
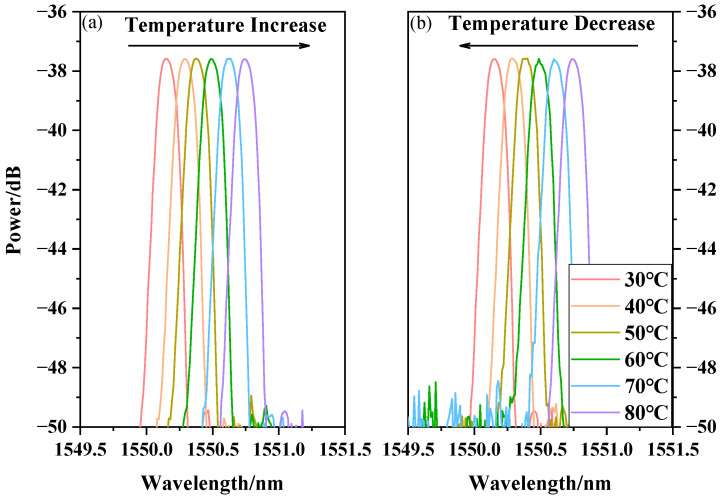
Temperature response with temperature increase and decrease. (**a**) Temperature Increase; (**b**) Temperature Decrease.

**Figure 13:**
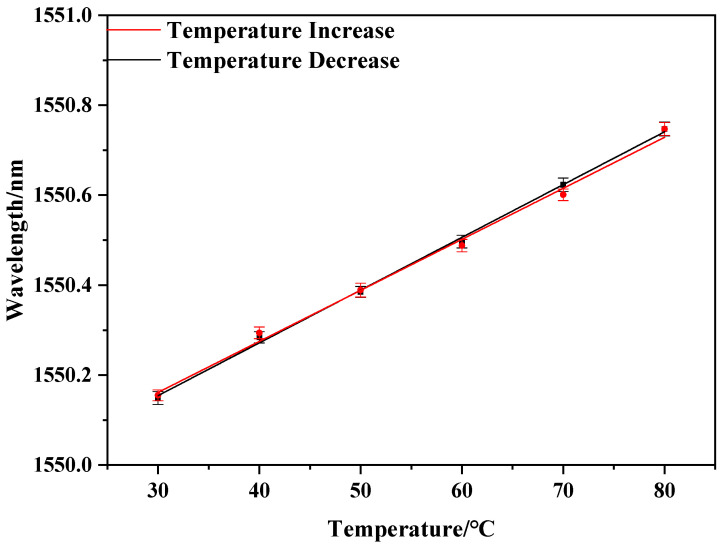
Temperature response fitting curve.

We implemented temperature experiments to verify whether the sensor could be used to measure temperature. The RH of the chamber was set at 55% RH and maintained for 30 min. The temperature increased from 30 °C to 80 °C, and the output spectra of OSA were recorded every 10 °C. Then, the spectra during the temperature decreasing process went from 80 °C to 30 °C. As observed in Figure 12a,b, the change of temperature caused a shift of the center wavelength of the FBG. In the processes of temperature increase and decrease, the relationship between the center wavelength of FBG and temperature is shown in Figure 13. The results indicate that the center wavelength shift is linear with temperature. The fitting lines of the response relationships in increasing and decreasing processes are y = 0.0118x + 1549.8 andy = 0.0114x + 1549.8, respectively. The slopes of the fitted lines indicate that the center wavelength of the FBG will shift 11.8 pm and 11.4 pm for every 1 °C change. The average value of the sensitivities was 11.6 pm/°C. 

### 3.3. Sensor Repeatability Test

**Figure 14:**
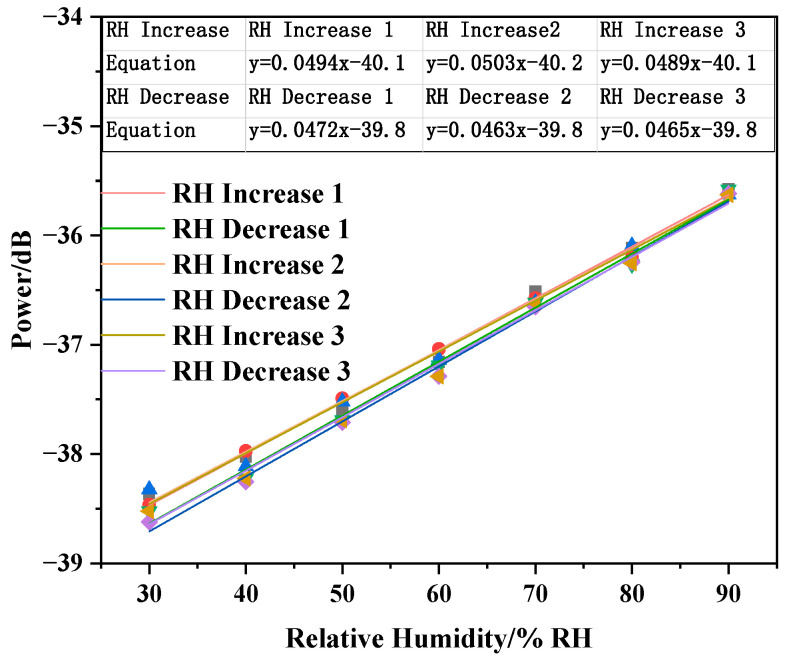
Sensor humidity response repeatability test fitting curve.

Ten days after the sensor test was completed, to demonstrate the performance repeatability and stability of the proposed sensor, we repeated the humidity response test three times at an interval of two days. The humidity response test results are presented in Figure 14. The measured sensitivities of the three humidity response tests were 0.0482 dB/%RH, 0.0483 dB/%RH, and 0.0476 dB/%RH. The experimental results indicate that after 10 days and the repeated tests, the sensor still worked normally with good repeatability. Moreover, the sensitivities of the test results are close to the original value, and the error was acceptable, indicating that the sensor had good stability and repeatability.

After obtaining the sensor’s humidity and temperature response features, we can get temperature and humidity information by tracing the wavelength and power of Bragg peak. The scanning speed of the spectrometer in high-resolution mode is relatively slow. In practical applications, the optical fiber demodulator with a scanning frequency of several kHz can be used to achieve high-speed real-time measurement.

## 4. Conclusions

In summary, an optical fiber sensor that can measure temperature and humidity simultaneously using a tapered MMF cascaded with an FBG was designed. Electrospinning was introduced to as a replacement for the traditional method of coating a humidity-sensitive film on optical fiber sensors. When CNTs were added to PVA electrospinning nanofibers, the humidity sensitivity was improved. Moreover, the mechanical strength could be improved. The experimental results indicated that the humidity sensitivity of the designed sensor with PVA/CNTs composite nanofiber film is 0.0483 dB%/RH, which was 31.16% higher than that of the sensor with the PVA nanofiber film. The addition of CNTs effectively eliminated the cracks on the electrospinning nanofibers and made them more uniform and smoother. The temperature sensitivity of the sensor was obtained by monitoring the shift of the center wavelength of the FBG. This nanofiber humidity-sensitive film prepared by electrospinning showed a satisfactory humidity response with a special 3D nanostructure and extensive application prospects.
